# Is it acceptable to contact an anonymous egg donor to facilitate diagnostic genetic testing for the donor-conceived child?

**DOI:** 10.1136/medethics-2018-105322

**Published:** 2019-06-12

**Authors:** Rachel Horton, Benjamin Bell, Angela Fenwick, Anneke M Lucassen

**Affiliations:** 1 Faculty of Medicine, Clinical Ethics and Law, University of Southampton, Southampton, UK; 2 Faculty of Medicine, Southampton Medical School, University of Southampton, Southampton, UK

**Keywords:** ethics, genetic screening/testing, informed consent, genetic information, reproductive medicine

## Abstract

We discuss a case where medically optimal investigations of health problems in a donor-conceived child would require their egg donor to participate in genetic testing. We argue that it would be justified to contact the egg donor to ask whether she would consider this, despite her indicating on a historical consent form that she did not wish to take part in future research and that she did not wish to be informed if she was found to be a carrier of a ‘harmful inherited condition’. We suggest that we cannot conjecture what her current answer might be if, by participating in clinical genetic testing, she might help reach a diagnosis for the donor-conceived child. At the point that she made choices regarding future contact, it was not yet evident that the interests of the donor-conceived child might be compromised by her answers, as it was not foreseen that the egg donor’s genome might one day have the potential to enable diagnosis for this child. Fertility consent forms tend to be conceptualised as representing incontrovertible historical boundaries, but we argue that rapid evolution in genomic practice means that consent in such cases is better seen as an ongoing and dynamic process. It cannot be possible to compel the donor to aid in the diagnosis of the donor-conceived child, but she should be given the opportunity to do so.

## Introduction

Genomic testing is blurring the boundaries between clinical tests and research. Children with undiagnosed genetic conditions are now frequently offered the chance to access genome sequencing via hybrid clinical research initiatives such as the 100,000 Genomes Project, aiming to establish why health problems have arisen.^1^ Such testing is ‘clinical’ in the sense that it is offered by National Health Service (NHS) healthcare professionals aiming to reach clinical diagnoses, but it is inseparably linked to research as in the process of such testing, new genetic conditions may be discovered or characterised.^2^ Genome sequencing works by detecting the approximately 100,000 rare variants that each person has in their genome.^3^ These are filtered down aiming to find the cause for a person’s health problems, although based on current scientific knowledge, it is often hard to work out which of the variants might be causing disease.^4^ Comparing an unwell child’s genome sequence with that of their biological parents (trio testing) can substantially increase the likelihood of diagnosis, as it makes it much easier to shortlist possible disease-causing variants because inherited variants can often be ruled out.^2 5^ By requiring samples from healthy relatives, the process draws people into genomic investigations who would not historically have been conceptualised as patients.

Progress in fertility treatments is also generating new ethical issues. The practice of egg donation began in the early 1980s, involving obtaining eggs from a donor woman, fertilising them with sperm and implanting the embryos created in the uterus of a recipient woman.^6^ Donors undergo hormone injections to stimulate egg production, followed by a procedure to collect the eggs.^7^ The process raises a number of ethical questions including how to compensate donors, and how to ensure that their medical needs and preferences are not overlooked in an attempt to fulfil the recipient’s desire for a child.^8^ In the UK, the Human Fertilisation and Embryology Authority oversee egg donation. Prospective donors need to provide information about their personal and family history and may undergo limited genetic testing to check their carrier status for various common genetic conditions.^9^

## Case

A child born as a result of egg donation presented with features suggestive of a rare genetic condition. Routine NHS diagnostic genetic tests were unable to identify a genetic cause for their difficulties, but the child was potentially eligible for further diagnostic testing in the context of the 100,000 Genomes Project. This involves trio genome sequencing, where the genome sequence of a child is compared with the genome sequences of both of their biological parents. Samples from the biological father and the child were readily available.

The fertility clinic that had facilitated the egg donation was asked whether it would be possible to approach the egg donor regarding potential participation in the 100,000 Genomes Project, with the aim of finding a diagnosis for the child that had been conceived using her donated egg. The fertility clinic declined to contact the egg donor on the basis that she had indicated on her consent form that she did not wish to be contacted about future research. She had also indicated that she did not wish to be notified if it was found that she had a previously unsuspected genetic disease or if she was found to be a carrier of a ‘harmful inherited condition’. This meant that the donor-conceived child could not have trio genome testing, reducing the chance of finding a genetic diagnosis for their difficulties (with the associated potential benefits of a clearer prognosis and more informed future care).

## Is it acceptable to contact the egg donor?

We argue that it would be ethically acceptable for the fertility clinic to contact the egg donor in order to explore the possibility of trio genome testing for the child conceived from her donated egg. We discuss our rationale for thinking that contact would be ethically justifiable in this situation.

### Reaching the limits of consent

At the core of this case is the question of what constitutes valid consent, or indeed valid refusal of consent, and how far it can take us. Did the questions asked of the egg donor at the time of donation encompass the issue of future contact to facilitate diagnosis for a child born as a result of the donation? Even if they did, what weight should we give to the ticking of boxes on a form many years ago—should we follow the choices apparently made at that point or is it acceptable to consider that the situation may have evolved such that current reality might supersede those previous abstract choices?

The concepts of consent that often imbue medical practice have largely been shaped around decisions with relatively limited potential consequences, such as whether or not to take a particular medication, or whether or not to have surgery. Professional guidance such as the GMC document ‘Consent: patients and doctors making decisions together’^10^ and cases where the issue of consent has been debated via the courts^11^ have tended to centre around the provision of adequate information in helping patients make binary, time-locked choices with a limited number of possible outcomes. From a legal perspective, the purpose of consent appears to be as a vehicle for respecting individual autonomy: what matters is the ability to exercise choice, rather than what the choice might be.^12^

There is little professional guidance as to how we should approach the notion of consent in situations where by virtue of a near infinite number of possible outcomes or because of unpredictable evolution in technology, no one can truly understand, retain and weigh all the relevant information in the depth that we might historically consider necessary for valid consent. ‘Broad consent’ is gaining increasing traction as a feasible option, coming to the fore in situations where providing specific information regarding all possible implications of a decision would be overwhelming or impossible, for example, for biobank participants^13^ or people having genomic testing.^14^ However, this has largely been to facilitate open-ended study or investigations and there has been little debate about what would constitute a valid refusal of broad consent.

Did the questions asked of the egg donor at the time of donation sufficiently cover the possibility of future contact to help make a diagnosis for any children born as a result of the donation? Details as to exactly what discussions she had when she donated her eggs are impossible to obtain from medical records—written consent forms provide some idea, but cannot perfectly reflect the nuances of the consent conversation and to what extent particular decisions were context-dependent. The egg donor indicated that she did not want to be contacted about future research, but in the genomic era, the distinction between clinical and research testing is becoming increasingly artificial. If ‘research testing’ is the only avenue left to achieve a clinical diagnosis, we think that this contact can legitimately be viewed as more in the realms of healthcare than research, though made more complex by the fact that any resulting medical benefits would pertain to the donor-conceived child, rather than the egg donor. Innovative technologies transgress ‘research’ and ‘clinical treatment’ categories, representing a space where both may co-exist,^15^ and the difficulty in categorising genomic testing as one or the other makes it difficult to interpret the egg donor’s wishes. When she chose to decline contact about future research, was she picturing ‘pure’ research where any impact on healthcare might be far removed and nebulous, or was she also considering hybrid clinical research endeavours such as the 100,000 Genomes Project, where the use of new technology might lead to immediate, tangible clinical benefit?

Similarly, the egg donor indicated that she did not wish to be contacted if it was found that she had a previously unsuspected genetic disease or if she was found to be a carrier of a ‘harmful inherited condition’. But in this case, the issue is that her biological child has a likely genetic disorder that needs explanation—as a side effect of testing, it might transpire that the egg donor is a carrier of a ‘harmful inherited condition’, but this would not be the intention of doing the test. Should we cling on to the fact that the egg donor ticked a box on a form saying that she did not want to find out information on carrier status as sufficient reason not to contact, if the primary reason for testing would be to diagnose the donor-conceived child, not to determine the egg donor’s carrier status? There are also potential medical benefits to the donor that should be considered: for some X-linked conditions, it is possible that she would benefit from medical interventions or screening herself.^16^ It is unlikely that this possibility was explored at the time of the egg donor’s initial consent conversation, so she may be unaware of the potential benefit of receiving such information. On a broader level, people who have been born or who have become parents through her egg donation may also have an interest in such information, as might prospective recipients if any of her donated eggs are still available.

Even if we consider that the egg donor’s choices covered the possibility of contact in this instance and indicated a preference against it, what weight should this have some years down the line? Should she be given an opportunity to consider the choice again? Recent research indicates that participants in the 100,000 Genomes Project sometimes have an inaccurate recollection of whether or not they chose to be informed of, for example, additional findings (genetic findings that might be relevant to their health but that would not account for the clinical condition that led to them joining the project).^17^ In the situation of egg donation, where people also have to take in a lot of information, it is easy to see how decisions about re-contact may seem small in the context of a greater decision about whether or not to donate and may be taken quickly without in-depth reflection about the potential consequences.

In other cases where potentially relevant theoretical decisions have been made some years ago, good practice would involve checking that the decision still holds. For example, if a patient has made an advanced decision to refuse a particular treatment, but at some point later, the treatment becomes a medically appropriate option, we would expect the patient’s medical team to revisit the decision with the patient if they still have the capacity to decide.^18^ There is no reason to think that the egg donor would now lack capacity, so we think it would be appropriate to give her the opportunity to consider whether to participate in genetic testing to help reach a diagnosis for the donor-conceived child. If her historical consent form had recorded ‘yes’ to potential future research and provision of genetic information, she would still have been allowed to say ‘no’ when asked whether she would now provide a DNA sample. Why does initially ticking the ‘no’ box automatically exclude her from any future decisions, even when they could not have been foreseen at the time of the initial consent conversation, and are potentially relevant to her own health?

We can stick to a rigid notion of boxes ticked on a consent form as a binding decision that makes any further discussion impossible. But in light of evidence that people may not recollect what choices they made when consenting to complex interventions,^17^ we argue that it is inappropriate to consider old consent forms as ‘trump cards’ that shut off other possible approaches that may not have been foreseen at the time consent was obtained. The form itself is not the consent—it is just a snapshot intended to reflect the consent dialogue that happened at the time. It may usefully inform future discussions, but to fetishize the form itself as having the final word in any question relating to a previous decision undermines the dynamic and collaborative nature of the consent process.

The question then arises as to what purpose written consent forms do serve. We think that they have value in that they may indicate preferences, highlight issues that need consideration and give insight into what discussions have previously taken place. We are not arguing that we should ignore them, but we dispute the idea that they should be taken as gospel. The wishes documented on a consent form are relevant to decision-making about subsequent actions, but they need to be considered in context and potentially weighed with and against other factors. For example, we might weigh the egg donor having ticked a box saying she did not want to be contacted about future research, against the likelihood that she meant pure research as opposed to clinical research initiatives, and the likelihood that if she knew the medical benefit that might entail to the donor-conceived child she would make the same decision.

### Should having a biological child curtail the choices you can make regarding future contact?

Another issue raised by this case is how the rights and interests of the egg donor interact with those of the child conceived from her donated egg. At a molecular level, they share half of their genetic information, so decisions regarding genetic testing in one inevitably impinge on the other. The possibility of future contact is embedded into the donor ‘contract’—when donor-conceived children reach 18, they can ask for their donor’s name, date of birth and last known address (provided that the donation was made after April 2005).^19^ Legally, the right of donor-conceived people to obtain information (although non-identifying) about their donor was recognised in UK law in *Rose v Secretary of State for Health* in 2002, when the High Court ruled that Article 8 of the European Convention on Human Rights included a right to know details about one’s identity, including information about biological parents.^20 21^

In the case that we describe, the rights of the donor-conceived child and of the egg donor are potentially in conflict. On the one hand, if the egg donor participates in genetic testing, it may help reach a diagnosis for the donor-conceived child, which would help them access better information and support regarding their genetic condition. On the other hand, the impact on the egg donor is more uncertain. She may perceive the contact as an unwelcome intrusion and an invasion of her privacy. She may not want to know that a child was conceived with her donated egg or that the child has a potential genetic condition, and that if she has other children they may also have a chance of being affected. However, it is also possible that the egg donor will find this information useful, for example, if she has gone on to have a child with a currently undiagnosed genetic condition, where information on the donor-conceived child could help all parties reach a diagnosis. She may also want to help the donor-conceived child reach a diagnosis for altruistic reasons. When she donated an egg, she had to accept the possibility of contact in the future from adults born because of her donation. We acknowledge that there is a difference in learning about the existence of a donor-conceived small child relative to the existence of an 18-year-old, but we argue that in this case, the age of the child does not make a significant difference to the ethical acceptability of contacting her. Putting off the contact until the age of 18 would delay a clear benefit to the child of improved diagnostic testing, based on an uncertain premise that the egg donor would accept finding out retrospectively that her donation had led to the existence of a child, but would be unwilling to learn this more contemporaneously. Moreover, the egg donor would not be contacted directly by the donor-conceived child in this circumstance, but by the fertility clinic that facilitated the donation.

In order to facilitate appropriate medical care for the donor-conceived child, we think it would be justifiable for the fertility clinic to approach the egg donor to ask the question of whether she would consider participating in genetic testing to help reach a diagnosis for the child born from her donation. Refusing to contact her based on a consent form that referred to her alone fails to consider that the interests of the donor-conceived child are inevitably dependent on the decision taken.

### Egg donors as patients

So far, we have argued that it may be appropriate to contact the egg donor to facilitate genetic testing for the donor-conceived child. This is both in view of the benefit that her genetic information could provide in diagnosing the child, and because it is not clear that she ever expressed a view regarding what she would have wanted to happen in the instance that by providing a DNA sample she could help the medical care of the donor-conceived child. Here, we consider whether there are any additional issues that we need to consider by virtue of the fact of her being an egg donor.

The way that egg donors are conceptualised by the healthcare community is unclear: previous research suggests that in some situations egg donors may be vulnerable to mistreatment or may be seen as ‘spare parts’, rather than being conceptualised as patients in their own right.^22^ Egg donation involves healthy women being exposed to medical risk in order to benefit another person, by enabling them to have a child. It has been argued that egg donation should therefore be held to a particularly high standard of consent, given the lack of benefits to the donor and the risk they undertake in becoming a donor.^7^ Historically, egg donation consent processes have often been notoriously inadequate.^23^ Research in the USA looking at the experiences of previous egg donors found that only 21% reported that they thought that there were serious psychological risks associated with egg donation before undergoing the procedure. Only 5% spontaneously reported awareness of the risk that a donor-conceived child might want to seek out their egg donor or that the egg donor might want to locate the child, and 6% reported awareness that they might be curious about the ‘end result’ of the donation.^24^

The question arises of how and whether this usefully informs decision-making regarding whether to contact the egg donor in the case we describe. We consider that it is important to consider these concerns in making contact with the egg donor and to be aware that contact from the fertility clinic is likely to be very unexpected. It is essential to consider carefully how much information to provide in any initial contact, especially in light of evidence that some egg donors who feel positively about having donated attribute this feeling at least in part to a hope that their donation has led to the birth of a healthy child.^24^ It is also important not to coerce the egg donor to take part in genetic testing if she chooses not to.

These concerns make the situation more complex, but we do not think that this complexity should preclude the egg donor having an opportunity to make a choice as to whether to participate in genetic testing to help reach a diagnosis for the donor-conceived child. We recognise that it is important to have high standards for consent in the area of egg donation, but we argue that ongoing dialogue and clarification may be an acceptable element of that; high standards for consent does not mean conjecturing what the donor’s response might be to a question she was never meaningfully asked. For example, it would be possible to write to the egg donor in very general terms, explaining that genetic technology has advanced significantly since her donation and that occasionally it is helpful to have access to samples from biological parents in order to guide healthcare of any donor-conceived children. Of course, this may lead to her correctly conjecturing that a child was born from her egg donation who has a health problem, but there would still be room for her to retain uncertainty as to whether this is the case.

Instinctively, the decision to contact the egg donor may feel uncomfortable. We argue that this discomfort arises because we continue to rely on a narrow model of consent that is unsuited to technology such as genomic medicine and egg donation. There is an understandable concern that stepping away from written consent forms as being definitive risks being exploitative: does this mean that anything goes? Patient consent has never been the only factor that matters in medical decision-making—other issues come into play such as balancing benefits and risks, and aiming to use resources responsibly and fairly. For narrower interventions, these other factors tend to influence the decision of a clinician to offer an option in the first place, with patient consent being the final element that determines whether the option happens. For broader interventions, maybe we need to consider all these factors concurrently, with consent viewed as an ongoing process rather than the single moment that allows things to happen (or not). We need to move away from the idea that the way to solve all our problems is to be imaginative enough to think of all the possible outcomes of a choice and make sure that they are all down as boxes on a consent form. This might work for more tractable, time-locked decisions such as whether to start a statin or whether to get your knee replaced, but it is unsuited to more complex interventions occurring over many years, where the consequences may evolve as technology alters.

## Conclusions

In summary, consent plays a crucial role in medical decision-making, but we need to update how we think about it in light of progressing technology. It needs to be appropriate for the dynamic and shifting nature of complex interventions, especially those that occupy the space between clinical care and research. We are not suggesting that consent does not matter or that it no longer has a role, but we need to embed consent throughout medical decision-making, being updated and clarified where necessary, rather than seeing it as a once-and-for-all rubber stamp at the end of a process from which there is no going back. In the case we describe, we think that the fertility clinic should contact the egg donor to ask whether she would consider having genetic testing aiming to reach a diagnosis for the donor-conceived child. We consider that the questions that the donor was asked at the time of egg donation did not sufficiently encompass this possibility for us to be sure what she would want now, some years down the line. The potential benefit of this course of action to the donor-conceived child also needs to be given weight. We recognise the need for scrupulous consent practices in egg donation and the importance of avoiding coercion. However, we argue that in this instance, it would be ethically justifiable, and in keeping with high standards of consent, for the fertility clinic to contact the egg donor to facilitate medical care of the donor-conceived child.
